# The IL-10-producing NKT10 subset plays a critical role in preventing graft-versus-host-disease

**DOI:** 10.3389/fimmu.2026.1817755

**Published:** 2026-07-01

**Authors:** Abel Trujillo-Ocampo, Drew Boagni, Maison Grefe, Jelita Clinton, Hong He, Ling Yu, Dan Li, Qing Ma, Seon Hee Chang, Jin S. Im

**Affiliations:** 1 Department of Hematopoietic Biology & Malignancies, The University of Texas MD Anderson Cancer Center, Houston, TX, United States; 2 Department of Molecular and Cellular Oncology, The University of Texas MD Anderson Cancer Center, Houston, TX, United States; 3 Department of Therapeutic Discovery, The University of Texas MD Anderson Cancer Center, Houston, TX, United States; 4 Department of Immunology, The University of Texas MD Anderson Cancer Center, Houston, TX, United States; 5 Department of Stem Cell Transplantation & Cellular Therapy, The University of Texas MD Anderson Cancer Center, Houston, TX, United States

**Keywords:** bone marrow transplantation, GvHD, invariant natural killer T cells, NKT10, NKT17

## Abstract

Invariant natural killer T (iNKT) cells play a role in preventing graft-versus-host disease (GVHD) in bone marrow transplantation (BMT), but it is not known whether and how the NKT10 and NKT17 subsets prevent GVHD. Here, we investigated the anti-GVH effects of iNKT cell subsets in a major-MHC mismatched murine BMT in which BALB/c recipients (H-2^d^) received grafts from C57BL/6 (H-2^b^) donors. The graft consisted of bone marrow and T cells from Traj18^KO^ (iNKT cell deficient) mice supplemented with iNKT cells purified from donors with various genetic alterations of iNKT subsets. First, NKT17-enriched CD4^-^ iNKT cells showed anti-GVH effects similar to those of NKT2-enriched CD4^+^ iNKT cells, while NKT2/17 deficient iNKT cells failed to prevent GVHD, suggesting that either or both of these subsets have anti-GVH function. Furthermore, IL10^KO^ iNKT cells completely lost their protective effects, whereas IL17AFD^KO^ iNKT cells demonstrated partially abrogated anti-GVH effects, supporting the indispensable role of NKT10 in preventing GVHD after BMT. Using IL-17 fate-mapping mice, we demonstrated that NKT17 can trans-differentiate into NKT10 *in vitro* after antigenic stimulation under T regulatory type 1 (Tr1)-promoting conditions. In conclusion, NKT10 plays a role in preventing GVHD in BMT and NKT17 may contribute to the anti-GVH effects via trans-differentiation into NKT10.

## Introduction

Allogeneic stem cell transplantation (ASCT) is a curative immunotherapy for hematologic malignancies that achieves clinical efficacy through the graft versus leukemia (GVL) effect mediated by reconstituted donor T cells (1). While insufficient GVL effects lead to leukemia relapse, dysregulated donor T cells recognizing alloantigen can cause graft-versus-host disease (GVHD), the major complication after ASCT (2). Thus, optimal regulation of donor immunity is key to enhancing the GVL effects of donor T cells and preventing GVHD in ASCT.

CD1d-restricted invariant Natural Killer (iNKT) cells are rare but powerful regulatory cells known to exert a spectrum of functions ranging from immune-regulation to direct anti-tumor activity (3). Unlike classical regulatory T cells (Treg), iNKT cells are functionally (NKT1, NKT2, NKT9, NKT10, NKT17) and phenotypically (CD4^+^ vs CD4^-^) diverse, and this functional heterogeneity may contribute to their unique ability to control various forms of immune-dysregulation (4–6). Thus, iNKT cells have potential to play a master-regulator role to maintain a fine balance between the anti-GVH and GVL effects of donor T cells in ASCT.

iNKT cell functional subtypes are differentially distributed among CD4^+^ and CD4^-^ iNKT cells, and thus the CD4^+^ and CD4^-^ populations are associated with different functions (4–6). Human CD4^+^ iNKT cells express more FoxP3 and Th2 cytokines than CD4^-^ iNKT cells and may exert superior immune-regulation. On the other hand, CD4^-^ iNKT cells have higher expression of cytolytic granules and NK receptors and demonstrate increased cytolytic activity. Thus, they may have better antitumor effector function (6–9). Of note, in clinical studies of matched donor ASCT, a higher graft dose of total iNKT or NKT1/NKT17-enriched CD4^-^ iNKT cells was associated with better GVHD outcome (10, 11). This is an unexpected observation given the bona fide pro-inflammatory properties of CD4^-^ iNKT cells. In murine Bone Marrow Transplantation (BMT) model, the addition of FACS-isolated CD4^+^ iNK T cells to the donor graft suppressed murine GVHD via the expansion of Tregs (12–14). However, it is not known how effective their anti-GVH effects are compared with CD4^-^ iNK T cells. Lastly, an evaluation of the anti-GVH and anti-tumor effects of NKT1, NKT2, and NKT17 subsets in murine BMT reported that NKT2 and NKT17 displayed better immunosuppressive function, while NKT1 displayed superior anti-tumor effects without significant anti-GVH effects (15).

Recently, a murine IL-10-producing iNKT subset, NKT10, has been described in mice pretreated with the agonist glycolipid α−galactosyl ceramide (αGalCer) (16). This NKT10 subset is reported to play a regulatory role in the pathogenesis of obesity (17–19), diabetes (17, 18), experimental autoimmune encephalomyelitis (16), tumor progression (16), and colitis (20). However, it is not known whether and how NKT10 contributes to the iNKT-mediated anti-GVH effects after ASCT. Here, we investigated the potential role of NKT10 cells in preventing GVHD using a major MHC-mismatched murine BMT model.

## Materials and methods

### Animals

Animal experiments were conducted in compliance with the protocols approved by the MD Anderson Institutional Animal Care and Use Committee. Six to eight-week-old female BALB/c, C57BL/6, B6(Cg)-*Traj18^tm1.1Kro^*/J (Traj18^KO^), and B6.129P2-*Il10^tm1Cgn^*/J (IL-10^KO^) mice were purchased from Jackson Laboratories (Bar Harbor, ME), acclimatized for three weeks prior to the experimental procedures and maintained under specific pathogen-free conditions. IL-17RB^KO^/B6 mice were provided by Dr. Masaro Taniguchi and IL-17AFD^KO^/B6 mice were generated by crossing IL-17AF^KO^ mice (21) and IL17d^tm1Lex^ from Genetech-Lexicon Pharmaceuticals (22). IL-17Fate^+^ mice were generated by breeding Foxp3^RFP^ IL-10^eGFP^ IL-17A^Kata^ triple reporter mice with IL-17A Fate reporter mice (IL-17A^CRE^ Rosa26 STOP^fl/fl^ (R26^YFP^)), both of which were provided by Drs. Richard Flavell and Nicola Gagliani (23).

### Materials

T cell media (TCM) was made of Roswell Park Memorial Institute Medium (RPMI)-1640 supplemented with L-glutamine (Gibco, #11875-093), 10% heat-inactivated fetal calf serum (FCS) (Hyclone, #A-1115-L), 0.001 mg/ml gentamicin (Gibco, #15710-015), 0.1 mM nonessential amino acids (Gibco, #11140-050) and essential amino acids (Gibco, #11130-051), 10 mM HEPES buffer solution (Gibco, #15630-080), and 5.5 μM 2-mercaptoethanol (2-ME) (Gibco, #21985-023). The freezing media consisted of 45% TCM, 45% FCS, and 10% Dimethyl Sulfoxide (DMSO) (Sigma-Aldrich, #67-68-5). Phosphate-Buffered Saline (PBS, #10010-023) was purchased from Gibco (Grand Island, New York). Recombinant murine IL-2 (#575408), human IL-6 (#570806), and IL-23 (#574102) were purchased from Biolegend (San Diego, California). Recombinant TGFβ1 (7754-BH-100) was purchased from R&D (Minneapolis, Minnesota). Alpha-GalactosylCeramide (αGalCer, #867000) was obtained from Avanti Polar Lipids (Alabaster, Alabama) and solubilized in dimethyl sulfoxide (DMSO) at concentrations ranging from 100 to 500 μM. The following antibodies against specific targets were purchased from BioLegend (San Diego, California), BD Bioscience (San Jose, California), or R&D systems (Minneapolis, Minnesota): CD16/32 (2.4G.2), CD3e (145-2C11), CD4 (RMA-4), CD122 (TM-B1), RoRγt (G31-378), PLZF (R17-809), IL-10 (JESS-16E3), IL-17A (TC11-18H10), IL-4 (11B11), and IFNγ (XMG1.2). APC and PE-conjugated αGalcer/mCD1d tetramers were provided by the National Institutes of Health (NIH) Tetramer facilities. Anti-mouse CD4-microbeads (#130-104-451), and CD8α-microbeads (#130-104-076) were purchased from Miltenyi Biotech (San Jose, California). GolgiStop protein transport inhibitor (#554724), GolgiPlug protein transport inhibitor (#555029), Fixable Viability Stain 620 (#564996), and BD Cytofix/Cytoperm Fixation/Permeabilization Solution Kit (#554714) were purchased from BD Bioscience. The eBioscience FoxP3/Transcription Factor Staining buffer set (00-5523-00) was purchased from Invitrogen. The Ghost dye UV 450 (13-0868) was purchased from Tonbo Bioscience (San Diego, CA). Paraformaldehyde (PFA) 16% w/v aq solution (#043368) and LIVE/DEAD Fixable Red (L34971) were purchased from ThermoFisher (Waltham, Massachusetts).

### Cell preparation for donor grafts

First, bone marrow (BM) cells were prepared by flushing the murine tibiae and femurs from the donor wild type (WT) or Traj18^KO^ C57BL/6 (H-2^b^) with PBS. Conventional T cells (Tcon) were isolated from donor splenocytes using Magnetic Activated Cell Sorting (MACS) with anti-CD4 and CD8 microbeads according to the manufacture’s instruction. For the isolation of iNKT cells, splenocytes from eight to twelve-week-old donor C57BL/6 mice with various genetic backgrounds (WT, IL-17RB^KO^, IL-10^KO^, IL-17AFD^KO^, αGalCer pre-treated mice) were stained for iNKT cells with αGalcer/CD1d-PE tetramer, followed by MACS with anti-PE microbeads. Subsequently, enriched iNKT cells were stained for CD3 and/or CD4 and further purified on FACS Aria II cell sorter (BD Bioscience). To obtain iNKT cells from αGalCer-pre-treated mice, C57BL/6 mice were intraperitoneally injected once with 4μg αGalCer in DMSO/PBS or vehicle, and iNKT cells were purified 1 month after the αGalCer treatment as described above.

### Murine bone marrow transplantation

Animal experiments were conducted in compliance with the approved protocols of the MD Anderson Institutional Animal Care and Use Committee (IACUC). Briefly, recipient BALB/c mice (H-2^d^) between age of 8–12 weeks were irradiated with 800 cGy using the Cesium-137 irradiator on day -1, received donor grafts consisting of bone marrow (BM, 5x10^6^) or BM + conventional T cells (Tcon, 1x10^6^) from either WT or Traj18^KO^ C57BL/6 (B6, H-2^b^) ± iNKT cells (5x10^4^ or 1x10^5^) purified from WT, IL-17RB^KO^, IL-10^KO^, IL-17AFD^KO^, or αGalCer pretreated WT B6 mice on day 0. All recipient mice were monitored daily for survival and 2–3 times weekly for clinical signs of GVHD (skin (0:normal, 1: presence of scaling paws or tails, 2: presence of the denuded lesions), posture (0:normal, 1: kyphosis at rest, 2:kyphosis impairing movement), weight (0: weight loss <10%, 1: weight loss <25%, 2: weight loss ≥25%), activity (0:normal, 1: stationary more than 50% of the time, 2: stationary unless stimulated), fur (0:normal, 1: mild to moderate ruffling, 2: severe ruffling) as previously reported (24, 25).

### *In vitro* stimulation of iNKT cells

A single-cell suspension of splenocytes was prepared from IL-10^eGFP^ or IL-17Fate^+^ mice, and 2x10^6^ splenocytes were stimulated with 100 nM αGalCer in 200 μl TCM in one well of a 96-well plate containing murine IL-2 (mIL-2, 20 ng/ml) and additional Treg-promoting TGFβ1 (5 ng/ml) or Type 1 regulatory T cell (Tr1)-promoting cytokines IL-6 (25 ng/ml), IL-23 (25 ng/ml), and TGFβ (5 ng/ml). After the indicated time for culture, iNKT cells were stained for Ghost dye V450, CD3, CD4, and iNKTCR with αGalCer/CD1d tetramer in the presence of FcR blocker (αCD16/32), fixed with 2% PFA, and acquired using LSRFortessa X-20 (BD Bioscience, Franklin Lakes, NJ) or Cytek Aurora (Cytek Biosciences). The expression of IL-10^eGFP^, IL-17A^Katuschka^, and IL-17A Fate^YFP^ of iNKT cells was assessed using FlowJo v10 (FlowJo, LLC). The experiments were performed in quadruplicate.

### *In vivo* stimulation of iNKT cells

C57BL/6 mice were injected peritoneally with 4 μg of αGalCer in 200 μl PBS or PBS only. A month later, iNKT cells in the mice were restimulated *in vivo* by intraperitoneal injection of 1 μg of αGalCer in 200 ul PBS. After 90 min of *in vivo* restimulation, isolated splenocytes were further incubated in TCM containing GolgiStop (1:1500) and GolgiPlug (1:1000). Subsequently, the cells were stained for following surface markers: TCRβ CD4, CD8α, the iNKTCR with αGalCer/CD1d tetramer in the presence of the LIVE/DEAD Fixable Red dye and FcR blocker (αCD16/32) for 45 min at 4 °C, followed by washing. The cells were fixed with BD Fixation/permeabilization buffer for 20 min, followed by washing. The cells were stained for 45 min with BD Perm/Wash buffer containing various anti-cytokine antibodies. After washing, the cells were resuspended in PBS and subjected to acquisition using Cytek Aurora (Cytek Biosciences). The cytokine expression of iNKT cells was assessed using FlowJo v10 (FlowJo, LLC). A total of 4 mice were used per treatment group.

### Analysis of iNKT cell subsets

BALB/c thymocytes were stained for TCRβ, iNKTCR, and CD4 in the presence of the LIVE/DEAD Fixable Red dye and FcR blocker (αCD16/32) for 45 min at 4 °C, washed, and fixed/permeabilized with FoxP3/Transcription factor staining kit. The cells were then stained for RORγt and PLZF in the presence of FcR blocker (αCD16/32) with perm/wash buffer for an additional 45 min, followed by acquisition using BD LSRFortessa X-20. The expression of transcription factors was assessed using FlowJo v10 (Flowjo, LLC).

### Statistical analysis

The log-rank test was used to compare the differences in survival between the control and treatment groups, and Analysis of Variance (ANOVA) as well as Tukey’s Honestly Significant Difference (HSD) test was used to assess differences in variables between control and treatment groups. All statistical analyses were performed using GraphPad Prism 10 software. Statistically significant differences were deemed for any P-value less than 0.05.

## Results

**Figure 1:**
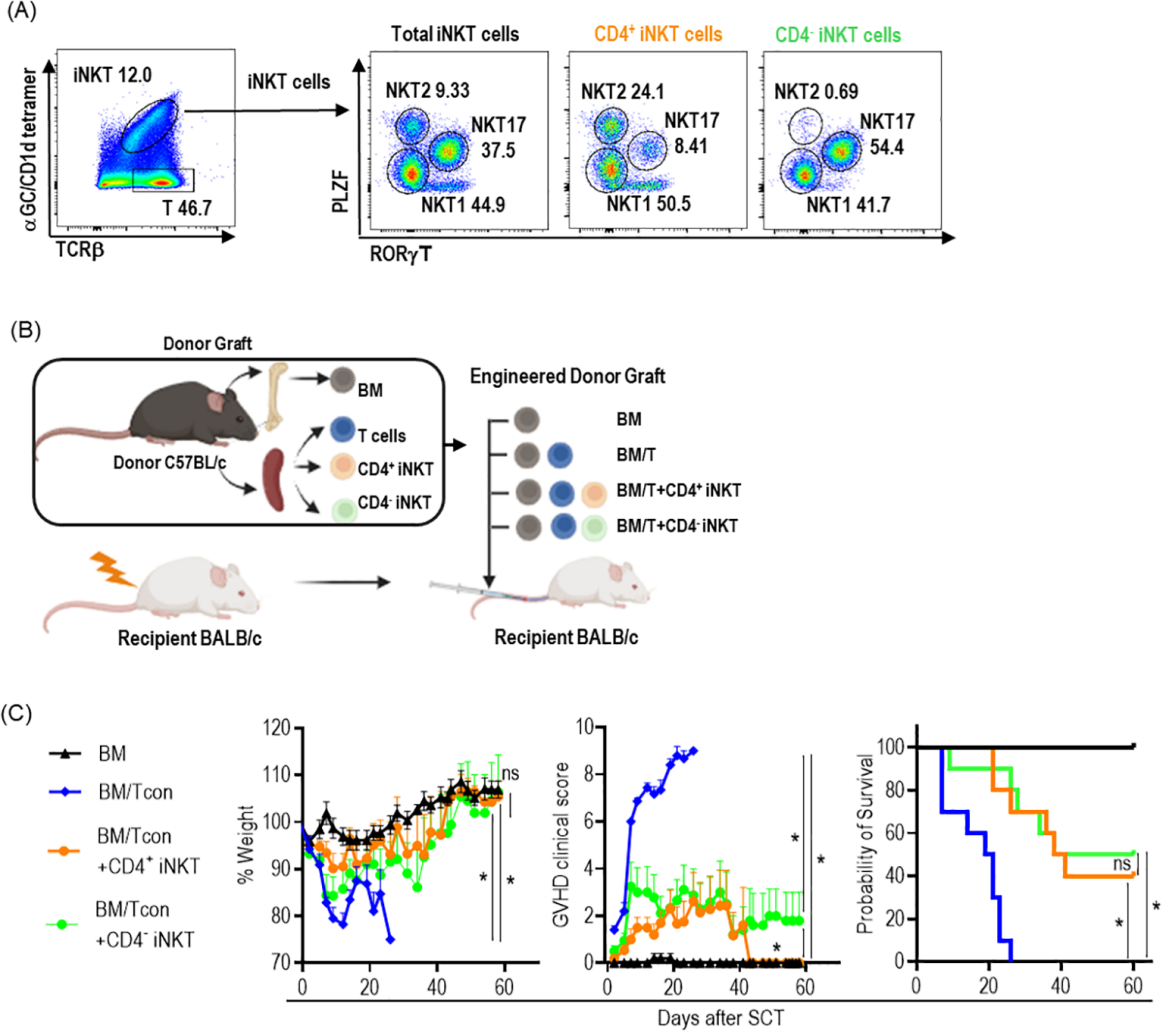
Both CD4^+^ and CD4^-^ iNKT cells similarly improve murine GVHD. **(A)** Distribution of NKT subsets in CD4^+^ or CD4^-^ iNKT cells. **(B)** BMT strategy: lethally irradiated recipient BALB/c mice (H-2^d^) received BM (5x10^6^) (n = 5) or BM + splenic T cells (1x10^6^) (n = 10) with or without CD4^+^ or CD4^-^ iNKT cells (5x10^4^) (N = 10 each) from donor C57BL/6 mice (H-2^b^). Subsequently, the recipient mice were monitored for clinical GVHD (weight, skin, fur, activity, and posture) and survival for 60 days. **(C)** The weight change, GVHD clinical score, and survival of the recipient mice. First, CD4^+^ iNKT cells were enriched with the NKT2 subset, while CD4^-^ iNKT cells were enriched with NKT17. NKT1 was similarly distributed among CD4+ or CD4- iNKT cells. Donor graft consisting of BM and splenic T cells from donor C56BL/6 mice led to the severe graft-versus-host disease (GVHD) and associated mortality in recipient BALB/c mice. The addition of CD4^+^ or CD4^-^ iNKT cells to the donor grafts significantly improved clinical GVHD and survival. Interestingly, NKT17-enriched CD4^-^ iNKT cells were as effective as NKT2-enriched CD4^+^ iNKT cells in improving GVHD-related mortality. The results were from one of two independent experiments. The log-rank test was used to compare the differences in survival between the control and treatment groups, and Analysis of Variance (ANOVA) as well as Tukey’s Honestly Significant Difference (HSD) test was used to assess differences in cumulative weight changes and GVHD scores between the control and treatment groups. * notes p<0.05. Ns refers “non-significant”.

In ASCT, both CD4^+^ and CD4^-^ iNK T cells have been shown to exert differing mechanisms of immune-regulation governed by the presence of distinct iNKT cell functional subsets (11–15, 26–30). For example, CD4^+^ iNKT cells are enriched for NKT2 while CD4^-^ iNKT cells are enriched for NKT17 (**Figure 1A**). However, their relative contribution in preventing GVHD has not been investigated in murine BMT. Therefore, we first validated the anti-GVH effects of CD4^+^ and CD4^-^ iNKT cells in a major MHC-mismatched murine bone marrow transplantation (BMT) model (**Figures 1B, C**). Here, lethally-irradiated BALB/c recipients received donor grafts from B6 donors consisting of BM plus Tcon ± highly purified CD4^+^ or CD4^-^ iNKT cells and were monitored for clinical GVHD and survival (**Figure 1B**). Both CD4^+^ and CD4^-^ iNKT cells were similarly effective in ameliorating murine GVHD when supplemented to the donor graft, mirroring the anti-GVH effects of human CD4^-^ iNK T cells (**Figure 1C**) (10, 31).

Recently, Maas-Bauer et al. reported that highly purified NKT2 and NKT17 cells controlled murine GVHD, while NKT1 showed better anti-tumor effects without significant anti-GVH effects (15). Specifically, NKT2 improved clinical GVHD and GVHD-related mortality more robustly than NKT17 (15). In our study, the absolute amount of NKT2 from NKT2-enriched CD4^+^ iNKT cells was lower than that of NKT17 in NKT17-enriched CD4^-^ iNKT cells as indicated in examples in **Figure 1A**. However, NKT2-enriched CD4^+^ iNKT cells (lower NKT2 number) showed similar anti-GVH effects compared with NK17-enriched CD4^-^ iNKT cells (higher NKT17 number). Thus, the results may confirm superior anti-GVH effects of NKT2 to NKT17 subset in line with the previous report (15).

**Figure 2:**
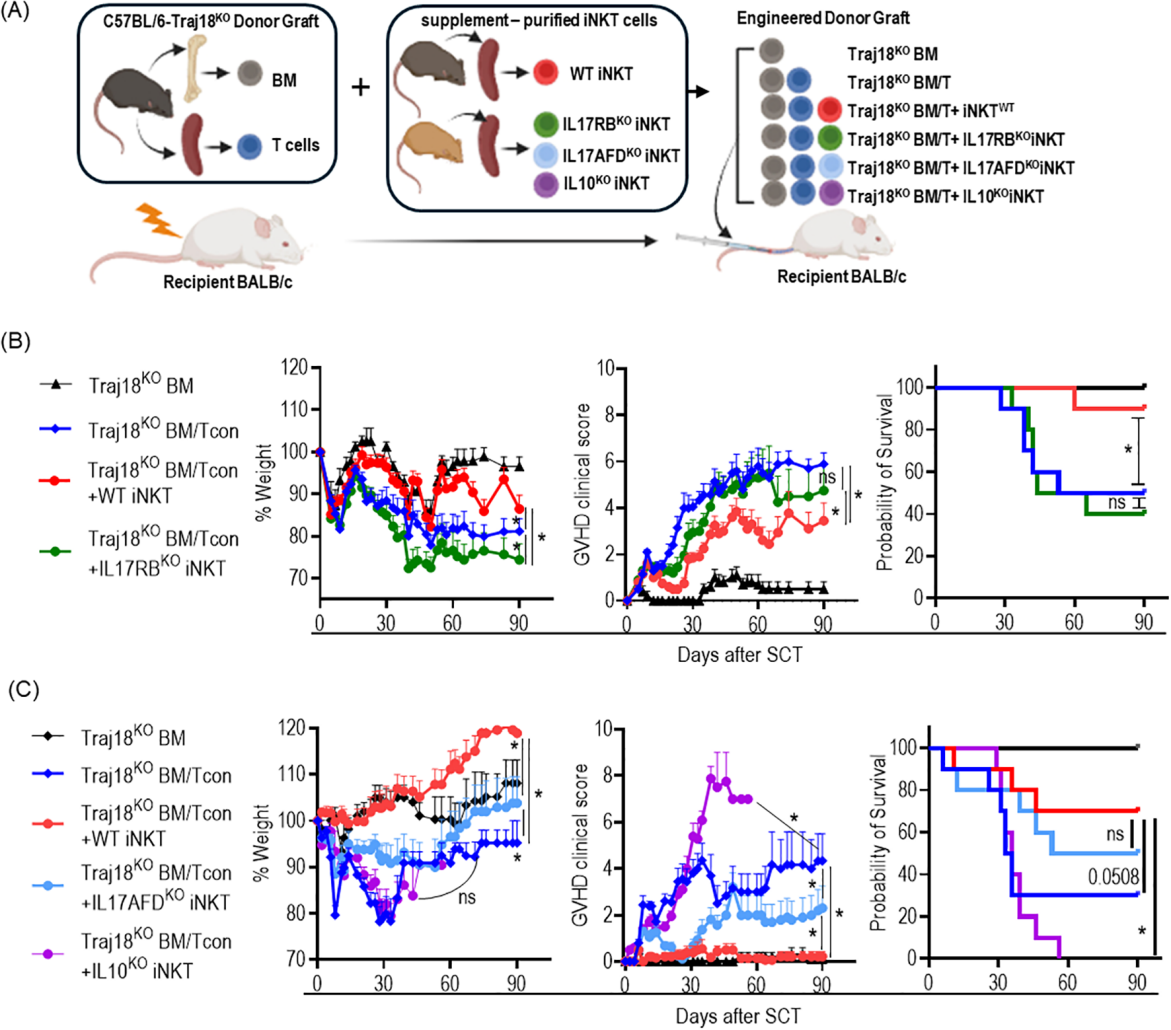
IL-10-producing iNKT cells play a role in preventing murine GVHD. **(A)** BMT strategy: lethally irradiated recipient BALB/c mice received BM (n = 5) or BM + splenic T cells (n = 10) from Traj18^KO^ C57BL/6 mice with or without iNKT cells (1x10^5^) from WT or IL-17RB^KO^ C57BL/6 mice (n = 10 each) **(B)**, or iNKT cells (1x10^5^) from WT or IL-17AFD^KO^ or IL-10^KO^ C57BL/6 mice (n = 10 each) **(C)**. Recipient mice were monitored for clinical GVHD (weight, skin, fur, activity, and posture) and survival over 90 days from the time of BMT. The weight change, GVHD clinical score, and survival of recipient mice of Traj18^KO^ C57BL/6 donor grafts containing WT or IL17RB^KO^ iNKT cells **(B)**, or WT or IL17AFD^KO^ or IL10^KO^ iNKT cells **(C)**. Donor graft consisting of BM and splenic T cells from Traj18^KO^ C56BL/6 mice induced clinically significant GVHD and GVHD-related mortality in recipient BALB/c mice. The addition of WT-iNKT cells to donor grafts significantly improved clinical GVHD and survival. The anti-GVH effects of iNKT cells were abolished when the NKT2 and NKT17 subsets were depleted from the donor iNKT cells via IL17RB^KO^. **(B)** The absence of IL-17A, IL-17F, or IL-17D from iNKT cells only partially abrogated the anti-GVH effects, while the deletion of IL-10 in iNKT cells not only abolished the anti-GVH effects of iNKT cells but also worsened clinical GVHD and GVHD-related mortality **(C)**. The results were from one of two independent experiments **(B)** or from single experiment **(C)**. The log-rank test was used to compare the differences in survival between the control and treatment group, and Analysis of Variance (ANOVA) as well as Tukey’s Honestly Significant Difference (HSD) test was used to assess the differences in cumulative weight changes and GVHD scores between the control and treatment groups. * notes p<0.05. Ns refers “non-significant”.

Independently, we confirmed the anti-GVH effects of NKT2 and NKT17 in murine BMT. Here we used donor grafts derived from Traj18(iNKT)^KO^ C57BL/6 donors, supplemented with iNKT cells isolated from wild-type or IL-17RB^KO^ C57BL/B6 mice (**Figure 2A**) (32). IL-17RB^+^CD4^+^ iNKT cells produce Th-2 cytokines (IL-4, IL-9, IL-10, IL-13), whereas IL-17RB^+^CD4^-^ iNKT cells produce IL-17A in an e4BP4-dependent fashion (32). Thus, IL-17RB^KO^ iNKT cells are deficient in the production of both Th2 and Th17-type cytokines and showed a complete loss of anti-GVH effects of iNKT cells when supplemented to donor grafts. This finding indicates that IL-17RB^+^CD4^+^ NKT2 and IL17RB^+^CD4^-^ NKT17 subsets are responsible for preventing GVHD after BMT (**Figure 2B**). In other words, the remaining NKT1 in IL-17RB^KO^ iNKT cells did not have significant anti-GVH effects, in line with the previous study (15).

e4BP4 can also regulate the production of IL-10 in CD4^+^ T cells and IL17RB^+^ iNKT cells (32, 33). Thus, we investigated the relative role of NKT10 to NKT17 in preventing murine GVHD. Here, we purified iNKT cells from IL-10^KO^ or IL17AFD^KO^ donor C57BL/6 mice and supplemented them with Trja18^KO^/B6 donor grafts (BM+T) for BALB/c recipients in murine BMT (**Figure 2C**). The deletion of IL-10 in donor iNK T cells obliterated their anti-GVH effects, while the absence of IL17AFD in donor iNKT cells only partially compromised the anti-GVH effects.

**Figure 3:**
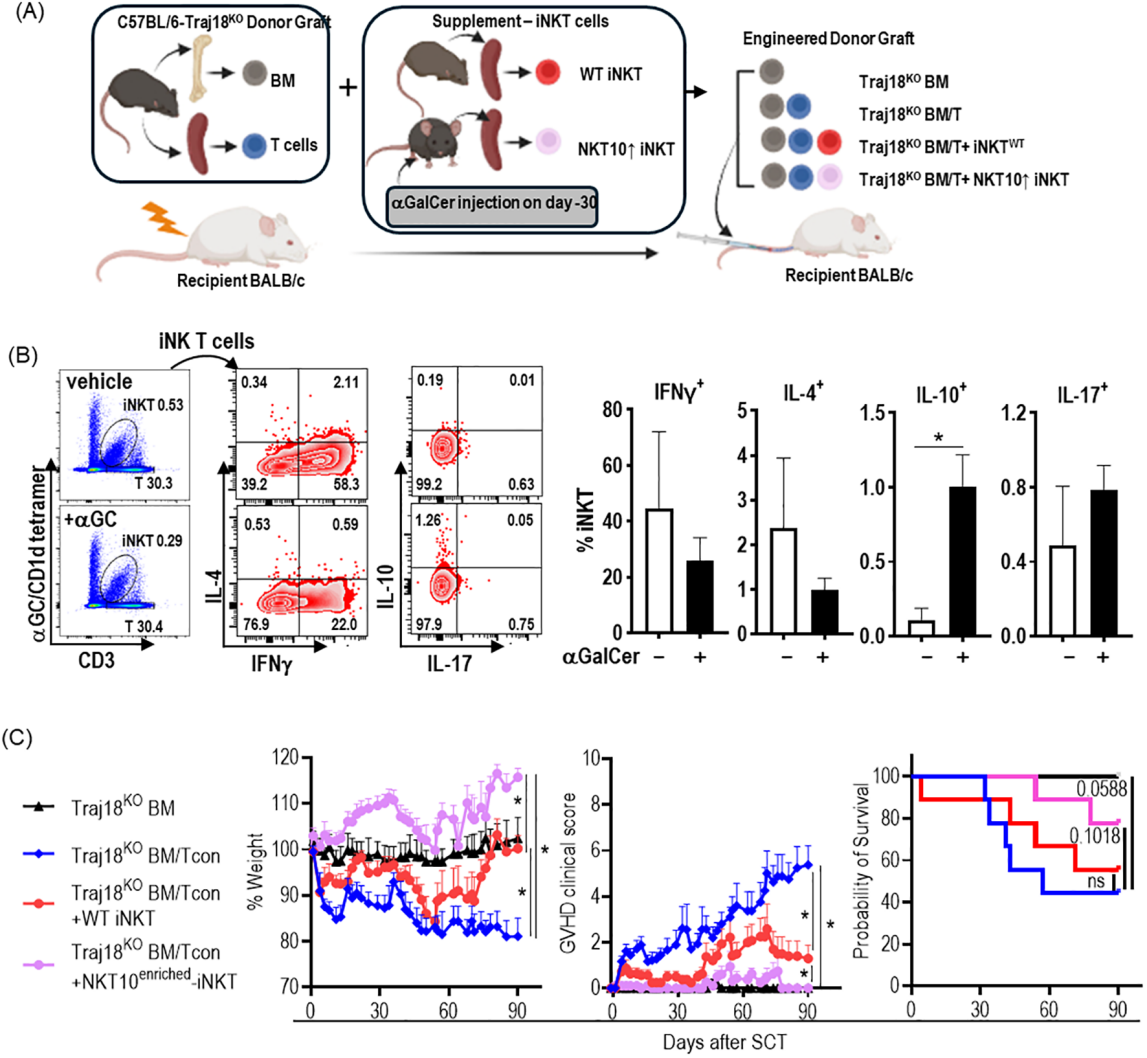
NKT10-enriched iNKT cells improve murine GVHD. **(A)** BMT strategy: lethally irradiated recipient BALB/c mice received BM (n = 5) or BM + splenic T cells (n = 9) from Traj18^KO^ C57BL/6 mice with or without iNKT cells (5x10^4^) from iNKT cells from C57BL/6 mice or NKT10-enriched iNKT cells from αGalCer-pretreated C57BL/6 mice (n = 9 each). The recipient BALB/c mice were monitored for clinical GVHD (weight, skin, fur, activity, and posture) and survival over 90 days from the time of BMT. **(B)** C57BL/6 mice (n = 4) received αGalCer (4 μg) or vehicle. A month later, iNKT cells from αGalCer or vehicle-treated mice were restimulated *in vivo* with the injection of αGalCer (1 μg), and splenocytes from these mice were prepared 90 min after *in vivo* restimulation and assessed for the presence of IFNγ, IL-4, IL-10, and IL-17 intracellularly. Results from one of two similar experiments were shown. **(C)** The weight change, GVHD clinical score, and survival of recipient BALB/c mice of Traj18^KO^ C57BL/6 donor grafts containing iNKT cells from C56BL6 donor mice or αGalCer-pretreated C56Bl/6 donor mice. The results were from one of two independent experiments. While both IFNγ^+^ and IL-4^+^ iNKT cells exhibited a trending decrease (p = 0.2364, 0.1330, respectively), there was a significant increase in IL-10^+^ iNKT cells, and a trend toward increase in IL-17^+^ iNKT cells (p = 0.1349) in the spleen of mice after treatment with αGalCer. Enrichment of the IL-10-producing NKT10 subset in donor iNKT cells improved clinical GVHD and GVHD-associated survival. The log-rank test was used to compare the differences in survival between the control and treatment group, and Analysis of Variance (ANOVA) as well as Tukey’s Honestly Significant Difference (HSD) test was used to assess the differences in cumulative weight changes and GVHD scores between the control and treatment groups. The Student’s *t*-test was used to compare differences between the two groups. The error bars represent mean ± standard deviation. * notes p<0.05. Ns refers “non-significant”.

To further confirm the anti-GVH effects of NKT10, we first enriched NKT10 in donor C57BL/6 mice with a single injection of αGalCer as previously reported (16), and then purified NKT10-enriched iNKT cells and supplemented them to Traj18^KO^/B6 donor grafts (**Figure 3A**). A single injection of αGalCer has been shown to increase murine IL-10^+^ iNKT cells in the spleen to an average of 5% (16). We also demonstrated that a single injection of αGalCer led to a significant expansion of IL-10^+^ iNKT cells and concurrent alteration of other NKT subsets such as a trending increase in NKT17 (p = 0.1349) and, reciprocally, a trending decrease in NKT1 and NKT2 (p = 0.2364, 0.1330, respectively) (**Figure 3B**). Thus, these αGalCer-pretreated iNKT cells consisted of altered iNKT subsets favoring immunosuppression and showed significantly improved clinical GVHD scores and a trend of improved survival (p = 0.1018) of the recipient mice (**Figure 3C**). Although the enriched NKT10 in αGalCer-pretreated iNKT cells may have significantly contributed to the improved anti-GVH effects, it may be difficult to accurately assess how altered NKT1, NKT2, and NKT17 subsets synergistically attributed to their anti-GVH effects of polyclonal iNKT cells.

**Figure 4:**
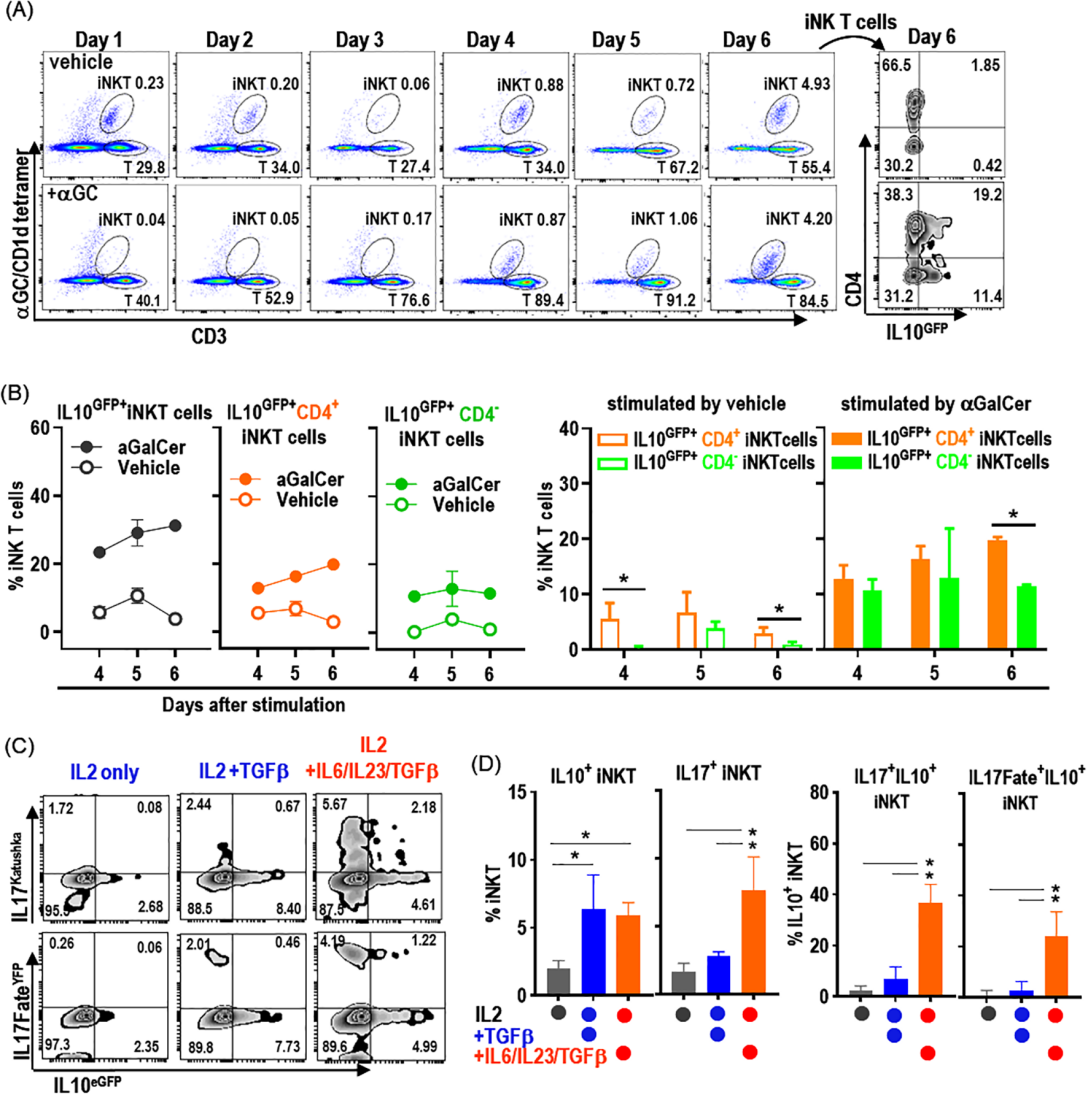
NKT17 trans-differentiates to NKT10. **(A, B)** 2x10^6^ splenocytes from IL-10^GFP^ C57BL/6 mice was stimulated with αGalCer (100 nM) and murine IL-2 (20 ng/ml), and the production of IL-10^GFP^ from CD4^+^ or CD4^-^ iNKT cells were assessed daily. Upon antigenic stimulation, iNKT cells were not detectable for the first 3 days, likely due to the downregulation of iNK-TCR and resurged from day 4. On average, 23.4% of iNKT cells expressed IL-10 on day 4, and expanded to 29.1% on day 5 and 31.3% on day 6. The percentage of IL-10^+^ iNKT cells was similar between CD4^+^ and CD4^-^ iNKT cells, especially on days 4 and 5, but IL-10^+^ CD4^+^ iNKT cells were significantly higher than IL-10^+^ CD4^-^ iNKT cells on day 6. **(C, D)** IL-17A Fate^+^ mice (L-17ACre.Rosa26STOP^f/f^YFPxIL-17A^Katushka^IL-10^eGFP^Foxp3^RFP^) were used to track NKT17, exNKT17 with a prior IL17 production, NKT10/NKT17 hybrid, and NKT10. Splenocytes from IL17A Fate^+^ reporter mice were stimulated with αGalCer and IL-2 under Treg-promoting conditions with additional TGFβ1 (5 ng/ml) or Tr1-promoting conditions with additional IL-6 (25 ng/ml), IL-23 (25 ng/ml), and TGFβ (5 ng/ml) for 4 days. The presence of IL-17A^Katushka^ (NKT17), IL-10^eGFP^ (NKT10), and IL-17^YFP^ (exNKT17) in iNKT cells was assessed. *In vitro* antigenic stimulation of iNKT cells from IL-17A Fate^+^ reporter mice showed a significant expansion of IL-10^eGFP^ iNKT cells under both Treg and Tr1-promoting conditions. However, the expansion of IL-17A^Katushka^ iNK T cells, IL-10^eGFP^ IL-17A^Katushka^ iNKT cells, and IL-10^eGFP^ IL17A^YFP^ iNKT cells were observed only under Tr1-promoting condition. Results were one of two independent experiments. Student’s t-test was used to compare the differences between the two groups. The error bars represent mean ± standard deviation. * notes p<0.05.

NKT17-enriched CD4^-^ iNKT cells exhibited similar anti-GVH effects to NKT2-enriched CD4^+^ iNKT cells (**Figure 1C**), and the loss of donor NKT10 was detrimental to the murine BMT (**Figure 2C**). Here, we investigated whether CD4^-^ iNKT cells regulate the anti-GVH effects via IL-10 production. To test this hypothesis, we first assessed whether CD4^+^ or CD4^-^ iNKT cells from IL-10^eGFP+^ splenocytes could produce IL-10 after antigenic stimulation *in vitro* and observed that CD4^-^ iNK T cells produced IL-10 at a level similar to that of CD4^+^ iNKT cells through day 3 from the stimulation but in a lesser degree than CD4^+^ iNKT cells on day 5 (**Figures 4A, B**). Thus, NKT17 subset in CD4^-^ iNKT cells may acquire the ability to produce IL-10 during GVHD. Similarly, this phenomenon has been described in conventional Th17 cells, which can transdifferentiate into Type 1 regulatory T cells (Tr1) after chronic inflammation (23).

Various markers have been used to identify iNKT subsets such as transcription factors (4), a combination of CD4 and CD122 (34), CD43 and ICOS (35), CD138 for NKT17 (36), or NRP1 for NKT10 (16). However, these markers may not be the best when studying the differentiation of iNKT subsets during activation as the expression may be altered upon activation. To investigate the potential transition of NKT17 to NKT10 upon activation, we utilized IL-17A Fate-mapping mice (IL-17ACre.Rosa26STOP^f/f^YFP) in which Cre-recombinases are transiently expressed under the IL-17A promoter and delete stop-cassettes to allow the permanent expression of YFP. In these mice, YPF marks T cells with prior and current production of IL-17 (37). We generated IL17A Fate^+^ mice (L-17ACre.Rosa26STOP^f/f^YFPxIL-17A^Katushka^IL-10^eGFP^Foxp3^RFP^) by crossing IL-17A Fate-mapping mice with IL-17A^Katushka^IL-10^eGFP^Foxp3^RFP^ triple reporter mice (23). This IL17A Fate^+^ mice allow us to characterize IL-17A^Katushka+^ iNKT cells as NKT17 that currently express IL-17, IL-17A-Fate^YFP+^ iNKT cells with a prior history of IL-17 production (exNKT17), and IL-10^GFP+^ iNKT cells as NKT10.

Splenocytes from IL17A Fate^+^ mice were stimulated with αGalCer and murine IL-2 under Treg-promoting conditions (TGFβ1), or Tr1-promoting conditions (IL-6, IL-23, and TGFβ) for 4 days. The presence of IL-17A^Katushka^(NKT17), IL-10^eGFP^(NKT10), IL-17-Fate^YFP^(exNKT17) in iNKT cells was assessed (**Figures 4C, D**). Both Treg and Tr1-promoting conditions significantly expanded NKT10 after antigenic stimulation, while only Tr1-promoting conditions increased NKT17. Interestingly, the fractions of the NKT10/NKT17 hybrid subset (IL-10^eGFP^+ IL-17A^Katushka^+) and NKT10/exNKT17 (IL-10^eGFP^+ IL-17A^Katushka^- Fate^YFP^+) were significantly increased under the Tr1-promoting condition. Our results suggest that NKT17s can transdifferentiate into NKT10s *in vitro* after antigenic stimulation under Tr1-promoting conditions mimicking chronic inflammation, suggesting a novel mechanism of immune-regulation by NKT17.

## Discussion

iNKT cells are thought to play a role in preventing GVHD in ASCT through IL-4 production (15, 38), elimination of alloantigen-presenting dendritic cells (31), and expansion of Tregs (12). Here, we present evidence that IL-10 production by iNKT cells may contribute to their anti-GVH effects. IL-10 is a potent anti-inflammatory cytokine that controls proinflammatory gene expression on various immune cells via IL-10/IL-10R/STAT3 pathways, modulating their immune-regulating function (39, 40). More specifically, IL-10 plays a critical role in the immunosuppressive function of classical Tregs (41). In addition, IL-10 produced by Tregs can enhance their expansion and suppressive function in an autocrine fashion (42, 43). Similarly, IL-10 produced by iNKT cells may help differentiate Tregs and reinforce their suppressive function to prevent GVHD. Previously, iNKT cells have been shown to facilitate donor Treg expansion in an IL-4-dependent manner in murine BMT (38), suggesting that iNKT cells mediate Treg-dependent anti-GVH effects through redundant pathways. However, the relative contributions of IL-4 by NKT2 and IL-10 by NKT10 to the anti-GVH effects of iNKT cells remain unanswered and require further investigation.

ASCT is a therapy in which recipients receive donor hematopoietic stem cells from either the bone marrow or peripheral blood that contain varying fractions of mature donor immune cells (1, 44). Thus, the early peripheral tolerance induction of mature T cells is likely a key to reducing GVHD risk in ASCT. Graft engineering with a precise control of donor T cells and Tregs is one promising approach to achieve early peripheral tolerance (45). However, iNKT cells will likely have advantages over Tregs as they can mediate additional GVL effects through innate natural killer-like properties, direct cytolysis of CD1d^+^ tumors, or promoting GVL effects of donor T cells (3, 46–49). Although we and others have shown that human iNKT cells in donor grafts mitigated GVHD in preclinical studies (31, 50), there is no clinical trial evaluating iNKT cell-based therapy to improve transplant outcome. This gap in clinical translation is due in large part to the paucity and functional heterogeneity of human iNKT cells. Here, we demonstrated that NKT10-enriched murine iNKT cells can be generated via antigenic stimulation under Treg and Tr1-promoting conditions *in vitro* (**Figure 4**). Likewise, one can potentially obtain NKT10-enriched human iNKT cells from *ex vivo* expansion via antigenic stimulation under similar conditions (50). Thus, it may be feasible to modify donor grafts with additional NKT10-enriched human iNKT cells at a minimum dose of 0.5-1x10^5^/kg, a number proven to be effective in improving ASCT outcomes (11, 26).

In summary, we provided novel insights into the regulatory role of NKT10 in BMT and the potential mechanisms through which NKT17-enriched CD4^-^ iNKT cells prevent GVHD. Further, we demonstrated that the NKT10, NKT10/NKT17, NKT10/exNKT17 subsets could be generated by antigenic stimulation under Tr1-promoting conditions *in vitro*. With further investigation, this could inform a clinical strategy of modifying ASCT donor grafts with NKT10/NKT17-like subsets generated by *ex vivo* expansion of human iNKT cells under Tr1-promoting conditions (50, 51). Thus, our findings can facilitate the clinical translation of iNKT-based cellular therapy for transplantation and other autoimmune diseases.

## Data Availability

The raw data supporting the conclusions of this article will be made available by the authors, without undue reservation.
